# Molecular detection of *Theileria* species*, Anaplasma* species*, Candidatus Mycoplasma haemobos, Trypanosoma evansi* and first evidence of *Theileria sinensis*-associated bovine anaemia in crossbred Kedah-Kelantan x Brahman cattle

**DOI:** 10.1186/s12917-021-02902-0

**Published:** 2021-07-18

**Authors:** Onyinyechukwu Ada Agina, Mohd Rosly Shaari, Nur Mahiza Md Isa, Mokrish Ajat, Mohd Zamri-Saad, Mazlina Mazlan, Azim Salahuddin Muhamad, Afrah Alhana Kassim, Lee Chai Ha, Fairuz Hazwani Rusli, Darulmuqaamah Masaud, Hazilawati Hamzah

**Affiliations:** 1 Faculty of Veterinary Medicine, Universiti Putra Malaysia, UPM, 43400 Serdang, Selangor Malaysia; 2 Department of Veterinary Pathology and Microbiology, Faculty of Veterinary Medicine, University of Nigeria, Nsukka, Enugu State 410001 Nigeria; 3 Animal Science Research Centre, Malaysian Agricultural Research and Development Institute, Headquarters, 43400 Serdang, Selangor Malaysia; 4 University Veterinary Hospital, Faculty of Veterinary Medicine, Universiti Putra Malaysia, UPM, 43400 Serdang, Selangor Malaysia; 5 Jabatan Perkhidmatan Veterinar, Pejabat KTS Zon Pahang Timur, 26700 Muadzam Shah, Pahang Malaysia

**Keywords:** Blood pathogens, PCR, Cattle, Haemato-biochemistry, Erythrocyte osmotic fragility, Phylogeny

## Abstract

**Background:**

Serious disease outbreaks in cattle are usually associated with blood pathogens. This study aims to detect blood pathogens namely *Theileria* species, *Anaplasma* species, *Candidatus Mycoplasma haemobos* and *Trypanosoma evansi*, and determine their phylogenetic relationships and haemato-biochemical abnormalities in naturally infected cattle.

**Methods:**

Molecular analysis was achieved by PCR amplification and sequencing of PCR amplicons of 18SrRNA gene of Theileria species, 16SrRNA genes of *Anaplasma* and *Mycoplasma* species, MPSP genes of *T. orientalis* and *T. sinensis*, MSP4 gene of *A. marginale*, 16SrRNA gene of *Candidatus Mycoplasma haemobos*, and RoTat1.2 VSG gene of *Trypanosoma evansi*, in sixty-one (61) clinically ill Kedah-Kelantan x Brahman cattle in Pahang, Malaysia.

**Results:**

A total of 44 (72.13%) cattle were infected with more than one blood pathogen. *Theileria* species was the blood pathogen with the highest molecular detection rate (72.13, 95% CI 59.83–81.81%). Nucleotide blast analyses of all sequences demonstrated high degree of molecular similarity (98–100%) in comparison with their respective reference sequences. Analysis of 18SrRNA gene sequences of *Theileria* species and 16SrRNA gene sequences of *Anaplasma* species revealed *Theileria sinensis* and *Anaplasma platys* respectively as additional species detected in these cattle. MPSP-PCR analysis was conducted for further confirmation of *T. sinensis.* The blood picture of eight infected cattle groups revealed poikilocytosis, anisocytosis, rouleaux formation and degenerative left shift. High mean erythrocyte fragility values were common in infected cattle groups. Anaemia of the macrocytic normochromic type and spherocytes were observed in the *T. evansi and Anaplasma platys + Theileria sinensis* double species co-infected cattle group. Normocytic normochromic anaemia was observed in the *T. sinensis* infected cattle group. Significant (*p* < 0.05) increases in serum liver and kidney parameters, total protein, globulin, total and unconjugated bilirubin and decreased albumin values were observed in the *T. evansi* infected cattle when compared to clinically healthy cattle.

**Conclusion:**

We present the first evidence of *Theileria sinensis*-associated bovine anaemia (TSABA) in Malaysian cattle. Because of the high occurrence of bovine theileriosis and detection of *A. platys*, there is an urgent need for appropriate preventive and control measures against these blood pathogens.

## Background

Theileriosis, anaplasmosis, hemoplasmosis and trypanosomosis are economically important vector-borne diseases of cattle, usually characterised by high morbidity and high mortality in clinical cases [1–5]. These diseases are ranked among the most common causes of economic losses in livestock industry [2]. The *Anaplasma, Theileria, Mycoplasma* (Haemotropic Mycoplasmas) *and Trypanosoma* species are the commonly found blood pathogens in Malaysian cattle [1, 6] and are transmitted by Ixodid ticks such as *Hemaphysalis sp.*, *Rhipicephalus sp.,* and *Amblyomma sp.* [7]. These blood pathogens have constrained cattle production in Malaysia [1], and affected cattle become severely anaemic, anorexic, feverish, jaundice, unthrifty and dehydrated. In addition, hyperproteinemia, leukopenia and thrombocytopenia have been reported in blood pathogen infected animals. Affected animals may abort or die in severe cases [8, 9]. A consistent feature of these diseases is that, following recovery from primary infection, affected cattle become asymptomatic and persistent carriers, acting as reservoirs of infection to susceptible or immunocompromised animals [9, 10]. These asymptomatic carriers show low levels of parasitaemia which is usually difficult to detect by microscopy [11]. Therefore, molecular techniques are usually more accurate in diagnosing subclinical or chronic infections [12, 13]. Trypanosomosis (Surra) is a mechanically transmitted disease of cattle and horses. It is of high endemicity in tropical and sub-tropical parts of the world. The causative agent is *Trypanosoma evansi*, an extracellular flagellated blood protozoan and the most widely distributed pathogenic trypanosome [14]. The acute form of the disease is characterised by anaemia with high mortality in susceptible animals, generalised loss of body condition, reduced fertility, poor immune response and death in protracted cases [15].

Bovine haemoplasmosis is caused by the haemotropic mycoplasmas namely *Mycoplasma wenyonii* and *Candidatus Mycoplasma haemobos* which are gram-negative, cell wall-less epierythrocytic bacteria found attached onto the surface of erythrocytes [16]. In affected herd, the bacteria cause severe anaemia, oedema and infertility [17, 18]. Anaplasmosis is caused by *Anaplasma marginale* and *A. centrale* in cattle [19, 20] and *A. platys* in dogs, ruminants and humans [21–23]. Intraerythrocytic inclusion bodies are found in blood smears of cattle infected with *Anaplasma* species. Also, anaplasmosis is mainly characterised by anaemia of the haemolytic type which may or may not be immune-mediated [24]. Anorexia, icterus, weight loss, decreased milk production, and dehydration are evident in susceptible animals [9]. Bovine theileriosis affects domesticated cattle and is associated with significant reduction in cattle productivity [25]. Theileriosis caused by *T. annulata* (tropical theileriosis) and *T. parva* (East coast fever) are of high pathogenicity [26], compared to the benign bovine theileriosis caused by the *T. orientalis/buffeli/sergenti* complex [26]. Oriental theileriosis is associated with low pathogenicity because *T. orientalis* schizont infected blood cells are not commonly observed in peripheral blood circulation in ruminants [27]. Recently, serious outbreaks of oriental theileriosis caused by the *T. orientalis* Ikeda type have been reported in the Asia-Pacific region [28, 29]. East coast fever caused by *T. parva* predominately affects cattle in Africa, but *T. annulata* has a world-wide distribution [2]. *T. parva* and *T. annulata* are the most pathogenic protozoa in animals [30], and their schizonts cause incomplete neoplastic transformation, proliferation and immortalisation of T cells [31]. *Theileria sinensis* which is a phylogenetically closely related member of *T. orientalis* complex is known to be one of the major causes of bovine theileriosis in China [32] and has been recently reported as one of the causes of benign bovine theileriosis in Malaysia [26]. It is of low pathogenicity in affected cattle [13].

In ruminants, haematology and serum biochemical analyses are relevant for detecting haematological disorders and pathological changes in vital organs of the body [33]. Results from such tests (complete blood count, liver and kidney function tests) provide valuable information that enables the clinician to arrive at a diagnosis, assess the efficacy of instituted therapy and monitor the disease process (static, progressing or regressing) [33]. An increase or decrease in haematology and serum biochemistry parameters have been reported in blood pathogen infections. The extent to which the haematology and serum biochemistry parameters are altered depends on several factors such as host immunity, age, infectious dose, virulence and infecting blood pathogen species [34]. *Anaplasma* and *Theileria* species infect and multiply inside the red blood cells, and during this intra-erythrocytic stage, intravascular or immune-mediated haemolysis can occur leading to anaemia [35]. Anaemia is a common finding in vector-borne diseases, and it is usually as a result of intravascular or extravascular destruction of the red cells. Anaemia of the macrocytic normochromic to macrocytic hypochromic (trypanosomosis) or normocytic normochromic (anaplasmosis and theileriosis) type is usually evident in affected animals [36]. Immune-mediated haemolytic anaemia has been reported in bovine anaplasmosis and theileriosis [24]. Osmotic erythrocyte fragility test is widely used as an aid to diagnosis of certain haematological disorders such as intravascular haemolytic anaemia, autoimmune diseases, spherocytosis, elliptocytosis and stomatocytosis [37, 38]. Evaluation of the membrane stability of erythrocytes under hypoosmotic stress is performed by lysis of erythrocytes in decreasing concentrations of hypotonic buffered saline solutions [39]. Erythrocyte osmotic fragility test is widely used as an aid to diagnosis of certain haematological disorders that involves alteration in erythrocyte membrane such as intravascular haemolytic anaemia, autoimmune diseases, hereditary spherocytosis, elliptocytosis and stomatocytosis [40]. Abnormalities in erythrocyte membrane also occur in bovine blood pathogen diseases such as babesiosis, trypanosomosis and anaplasmosis [8, 37, 41, 42]. Other factors such as nutritional status, hormonal imbalance, drugs and chemical intoxications, age, transportation stress could also increase the fragility of erythrocytes [43–45].

Diseases caused by blood pathogens have negative impact on the livestock industry in Malaysia. Therefore, it is essential to improve detection and surveillance of these blood pathogens [46]. Use of light microscopy for identification in stain blood smears are usually ineffective for differentiation of the species and detection of asymptomatic, presymptomatic and carrier animals with low parasitaemia. The use of molecular (PCR) based detection method has enabled better detection of these blood pathogens with high sensitivity and specificity [26, 30]. A need to determine the phylogenetic relationships and to have a better molecular taxonomic understanding of the infections caused by various species of commonly occurring blood pathogens in Malaysian cattle is required. Therefore, this present study provides the molecular characteristics of *Theileria* species, *Trypanosoma evansi, Candidatus M. haemobos* and *Anaplasma* species in Muadzam, Pahang Malaysia and ascertained the haemato-biochemical abnormalities and erythrocyte osmotic fragility in clinically ill crossbred Kedah-Kelantan x Brahman cattle infected with these blood pathogens.

## Methods

### Ethical statement

This study was conducted according to the guidelines of the Animal Care and Use Committee, Universiti Putra Malaysia, Animal Welfare Act (2014) AUP No. UPM/IACUC/AUP-R008/2020.

### Study population and blood sampling

Ten (10) mL each were obtained by coccygeal venipuncture from randomly selected 61 clinically ill and 17 clinically healthy Kedah-Kelantan x Brahman cattle of varying ages (1–11 years old) and sex (male and female) from a beef farm in Muadzam Pahang, Malaysia. The animals were reared in an oil palm plantation, and grazed forages under palm oil plantation with some mineral supplementation. The clinically ill cattle were physically examined, and clinical signs were noted before blood samples were obtained. The seventeen (17) clinically healthy cattle were selected based on absence of blood pathogens confirmed by throrough screening for blood pathogens by microscopic and molecular methods, absence of clinical signs of the disease, absence of tick infestation and moderate body condition. The blood samples were placed in a pre-labelled K2 EDTA vacutainer tubes for haematological analyses, heparin vacutainers for erythrocyte osmotic fragility test and plain vacutainers to harvest sera for biochemical analyses. The samples were then transported in an ice box to the Haematology and Clinical Biochemistry Laboratory, Faculty of Veterinary Medicine, Universiti Putra Malaysia. Whole blood samples for PCR were stored at − 80 °C prior to DNA extraction. Extracted genomic DNA was subjected to genera and species-specific PCR test for detection of these blood pathogens.

### Microscopic examination of stained thin-blood smears

Giemsa stained thin-blood smears were examined for possible detection and identification of blood pathogens with the aid of a light microscope (Nikon Eclipse 80*i*, Tokyo Japan).

### Determination of haemato-biochemical parameters

Haematology parameters such as haemoglobin concentration, red blood cell, total white blood cell and platelet counts were performed by an automated haemoanalysers (Advia 2120i Siemens-Healthineers, Malvern, USA). Packed cell volume was performed using the micro-haematocrit technique with the aid of haematocrit centrifuge (Haematokrit 20, Hettich Zentrifugen, Tuttlingen, Germany), and Hawksley micro-haematocrit reader (Hawksley, England). Sera were harvested using a portable table centrifuge (EBA 20 Hettich Zentrifugen, Tuttlingen, Germany). Serum sodium, potassium and chloride were determined using Siemens Xpand Plus Chemistry Analyser (Siemens Healthineers, Malvern, USA). Serum aspartate aminotransferase (AST), alkaline phosphatase (ALP), gamma glutamyltransferase (GGT), total bilirubin (TB), conjugated bilirubin (CB), inorganic phosphate (IP), urea, creatinine total protein (TP) and albumin (ALB), were done using BiOLis 24i Premium Chemistry Analyser (DiaSystem Scandinavia AB, Sweden). Globulin was calculated by subtracting the albumin value from total protein value. Albumin to globulin ratio was calculated by dividing the albumin value with the globulin value. Plasma protein was determined using a hand-held refractometer (Atago, USA) while icteric index was determined by comparing the colour of test plasma sample with a set of colour standards.

### Erythrocyte osmotic fragility test

Erythrocyte osmotic fragility (EOF) was determined by subjecting erythrocytes to varying concentrations of buffered saline solutions with slight modification [38, 47]. Briefly, 5 mL of different buffered saline concentrations of pH 7.4 ranging from 0.1 to 0.9% were pipetted into 5 test tubes arranged serially in a test tube rack. A set of 5 test tubes was used to analyse each sample. Whole blood (20 μL) was pipetted into each test tube and mixed thoroughly by inverting the test tubes several times to mix the content. The mixed blood was incubated at room temperature for 20 min. The content was mixed again before spinning at 1500 x g for 15 min. The supernatant was transferred into cuvette and read at an absorbance of 540 nm using a spectrophotometer (Tecan Biochem, USA). The percentage haemolysis was calculated with the following formula:

$$ \mathrm{Haemolysis}\left(\%\right):\left(\mathrm{Optical}\ \mathrm{density}\ \mathrm{of}\ \mathrm{test}/\mathrm{Optical}\ \mathrm{density}\ \mathrm{of}\ \mathrm{distilled}\ \mathrm{water}\right)\mathrm{x}100 $$

The EOF curve was obtained by plotting the haemolysis percentage against the different saline concentration. The result of the EOF is expressed as the saline concentration that caused 50% haemolysis, which is the mean cell fragility (MCF).

### Genomic DNA extraction

Genomic DNA was extracted from whole blood stored at − 80 °C using the DNeasy® Blood and Tissue kit (Qiagen, Hilden Germany) according to the manufacturers’ protocol. The eluted DNA was stored at − 20 °C until use. DNA concentration and purity were measured with a Nanodrop spectrophotometer (Tecan Infinite M200®, Austria). DNA samples with A_260_/A_280_ ratios between 1.7–2.2 were further analysed.

### Molecular identification by PCR

Polymerase chain reaction assay was done to amplify the partial gene fragments of *Anaplasma*, *Theileria* and *Mycoplasma* species using genus and species-specific primer sets (Table 1). Detection of RoTat 1.2 VSG gene for *T. evansi* was done using species-specific primer set (Table 1). Thermocyclic conditions and primers for *Anaplasma*, *Theileria* and *Mycoplasma* species were as specified by Parola et al. [48]; Sogin et al. [49]; and Su et al. [50] respectively (Table 1). All samples positive for 18S ribosomal RNA gene of *Theileria* species and 16S ribosomal RNA genes of *Anaplasma* and *Mycoplasma* species were further amplified by conventional PCR using species-specific primer sets (Table 1), targeting major piroplasm surface protein (MPSP) genes of *T. orientalis* and *T. sinensis*, major surface protein 4 (MSP4*)* gene for *A. marginale* and 16S ribosomal RNA of *C. Mycoplasma haemobos*. Thermocyclic conditions and primers for *A. marginale*, *T. orientalis*, *T. sinensis*, *T. evansi* and *C. M. haemobos* were as specified by Shkap et al. [51], Ota et al. [52], Liu et al. [53], Urakawa et al. [54] and Su et al. [50] respectively (Table 1). Each PCR run was performed in a final volume of 25 μL reaction in 0.2 mL PCR reaction tubes, comprised of 0.3 μL of 10 mM dNTP mix (dATP, dCTP, dGTP, dTTP), 1 μL of 10 μM of each primer, 5 μL of 5X Green GoTaq® flexi buffer (Promega Madison WI, USA), 5 μL of 25 mM MgCl_2_, 0.3 μL of 5 U GoTaq®G2 flexi DNA polymerase (Promega Madison WI, USA), 8.4 μL of water for molecular biology (Millipore corporation, Billerica MA, USA) and 100 ng of template DNA. The positive control DNA used in the PCR run consisted of a field strain confirmed by PCR and sequencing of the amplicon, and was obtained from the Veterinary Parasitology Laboratory, Universiti Putra Malaysia. Water for molecular biology (Millipore corporation, Billerica MA, USA) was used as a negative control. Both positive and negative controls were included in each PCR run. PCR amplification was performed using the BioER Little Genius® LED Thermal Cycler (Hangzhou Bioer Technology China). The gel was stained with RedSafe™ (iNtRoN Biotechnology, Korea) for 10 min, thereafter, amplified products were then analysed by electrophoresis on a 1.5% agar rose gel for 80 min at 80 voltage. The gel was visualised using a UV transilluminator (GeneDireX™, USA).

**Table 1:** Genus and species-specific primers, thermocyclic conditions and amplicon size of the various genes used for the detection and amplification of cattle blood pathogens. Thermocyclic conditions include initial denaturing (ID), denaturing (D), annealing (A), extension (E), and final extension (FE)

Blood Pathogens	Gene	Primer Sequences (5′-3′) Forward (F) and Reverse (R)	Thermocyclic conditions	Base pair (bp)	References
*Anaplasma* species	16SrRNA	EHR16SD - GGTACCYACAGAAGAAGTCC EHR 16SR -TAGCACTCATCGTTTACAGC	ID: 95 °C /5mins D: 95 °C /30s A: 55 °C /30s E: 72 °C /90s No of cycles: 40 FE: 72 °C /5mins	345	[47]
*Theileria* species	18SrRNA	F-ACCTGGTTGATCCTGCCAG R-GATCCTTCGCAGGTTCACCTAC	ID: 94 °C /5mins D: 94 °C /30s A: 60 °C /30s E: 72 °C /1 min No of cycles: 40 FE: 72 °C /5mins	1750	[48]
*Mycoplasma species*	16SrRNA	F-GGGAGCAAACAGGATTAGATACCCT R-TGCACCATCTGTCACTCTGTTAACCTC	ID: 94 °C /5mins D: 94 °C /30s A: 55 °C /30s E: 72 °C /1 min50secs No of cycles: 40 FE: 72 °C/10 mins	280	[49]
*Anaplasma marginale*	MSP4	F- CATCTCCCATGAGTCACGAAGTGGC R-GCTGAACAGGAATCTTGCTCCAAG	ID: 95 °C /5mins D: 95 °C /1 min A: 65 °C /2mins E: 72 °C /1 min No of cycles: 40 FE:72 °C/10 min	761	[50]
*Theileria orientalis*	MPSP	F- CTTTGCCTAGGATACTTCCT R- ACGGCAAGTGGTGAGAACT	ID: 94 °C /4mins D: 94 °C /1 min A: 63 °C /1 min E: 72 °C /1 min No of cycles: 40 FE:72 °C /7mins	776	[51]
*Theileria sinensis*	MPSP	Tsin F- CACTGCTATGTTGTCCAAGAGATATT Tsin R-AATGCGCCTAAAGATAGTAGAAAAC	ID: 94 °C /3mins D: 94 °C /1 min A: 56 °C /1 min E: 72 °C /1 min No of cycles: 40 FE:72 °C /7mins	887	[52]
*Trypanosoma evansi*	RoTaT 1.2 VSG	F-GCG GGG TGT TTA AAG CAA TA R-ATT AGT GCT GCG TGT GTT CG	ID: 94 °C/4mins D: 94 °C /1 min A: 59 °C /1 min E: 72 °C /1 min No of cycles:40 FE: 72 °C/5mins	205	[53]
*Candidatus Mycoplasma haemobos*	16SrRNA	F-GAGTTAGTTATTAAAGCTTTAT R-ATTCATGAGGTACTATCAGTTG	ID: 94 °C /5mins D: 94 °C /30s A: 55 °C /30s E: 72 °C /30s No of cycles: 40 FE:72 °C /7mins	279	[49]

### Sequencing of PCR products and BLAST analysis

Amplicons from each blood pathogen species with clear bright bands were selected for sequencing. They were gel extracted and purified using QIAquick gel extraction kit (Qiagen, Hilden, Germany) according to the manufacturers’ protocol. The amplicons were sequenced using the BigDye® Terminator v3.1 cycle sequencing ready reaction kit (Applied Biosystems, USA). Consensus sequences were obtained for all PCR products using BioEdit Sequence Alignment Editor Software (version 7.0.5.3). The resulting nucleotide sequences were analysed and compared for similarities with reference sequences from GenBank database, using the Basic Local Alignment Search Tool (BLAST) program (http://www.ncbi.nlm.nih.gov/BLAST.cgi). PCR amplicons of 18SrRNA and 16SrRNA gene found to be negative for *T. orientalis* and *A. marginale* respectively were sequenced using their respective genus primer sets.

### Sequence alignment and phylogenetic analysis

To determine the phylogenetic status of the various blood pathogen species identified in this study, a phylogenetic tree was constructed based on the MPSP gene of *T. orientalis* and *T. sinensis* for *Theileria* species, MSP4 gene of *A. marginale*, 16SrRNA gene of *C. M. haemobos* and RoTat1.2 VSG of *T. evansi*. Their sequences were supplemented with their respective reference sequences from GenBank and were aligned using ClustalW algorithm. After alignment, regions with gaps gap-free sites were used to construct phylogenetic trees using maximum likelihood method in MEGA software version X [55]. The reliability for the internal branches of maximum likelihood was assessed using 1000 bootstrap re-samplings and molecular distances estimated by the general time reversible model [56].

### Statistical analysis

All data, expressed as mean ± standard error of mean, were normally distributed (*P* > 0.05, Shapiro-Wilk’s test). Percentage occurrence of blood pathogens, both single and mixed infections, and 95% confidence intervals were calculated using Proportion test (*prop.test*) in Epitools software (https://www.epitools.ausvet.com.au/ciproportion). Chi-square test was used to determine the association between the presence of the blood pathogens and risk factors (gender and age of cattle). One-way multivariate analysis of variance (MANOVA), followed by Duncan multiple post hoc comparison test, was applied to study the effect of varying concentrations of saline solutions on erythrocytes. One-way analysis of variance (ANOVA) followed by Duncan post hoc comparison test were applied on the data generated from the haemato-biochemical parameters and mean cell fragility of the different groups of cattle. Mean cell fragility values were extrapolated from the erythrocyte osmotic fragility curves. Statistical analysis was performed using SPSS 25.0 (Chicago IL, USA). A Probability value < 0.05 was considered as statistically significant. The Kedah-Kelantan x Brahman cattle were later divided into eight categories based on the number of infecting blood pathogens; single species, double species co-infection, and triple species co-infection following PCR amplification and sequencing of species-specific genes*.*

### Accession numbers of nucleotide sequences

The nucleotide sequences of all genes have been deposited in the GenBank of the NCBI database and assigned accession numbers are listed in Table 2.

**Table 2:** GenBank accession numbers for the blood pathogens detected in this study

No.	Blood pathogens	Genes	GenBank Accession No.	Sequence length (bp)
1	*Theileria orientalis*	MPSP	MT184275 MT184276 MT184277 MT184278 MT184279	720 455 506 455 521
2	*Theileria sinensis*	18S rRNA	MT271898 MT271899 MT271900 MT271901 MT271902 MT271903 MT271904 MT271905 MT271906 MT271907 MT271908 MT271909 MT271910 MT271911	1693 1687 1663 1668 1670 1674 1661 1698 1698 1663 1682 1689 1651 1679
3	*Theileria sinensis*	MPSP	MT240816 MT240817	871 870
4	*Anaplasma marginale*	MSP4	MT173810 MT173811 MT173812 MT173813 MT173814	672 704 705 634 704
5.	*Anaplasma platys*	16S rRNA	MT259465 MT259466	349 348
6.	*Candidatus Mycoplasma haemobos*	16S rRNA	MT521752 MT521753	248 253
7	*Trypanosoma evansi*	RoTaT 1.2 VSG	MT514513 MT514514	203 204

## Results

### Clinical signs and microscopic examination of thin blood smears

**Fig. 1:**
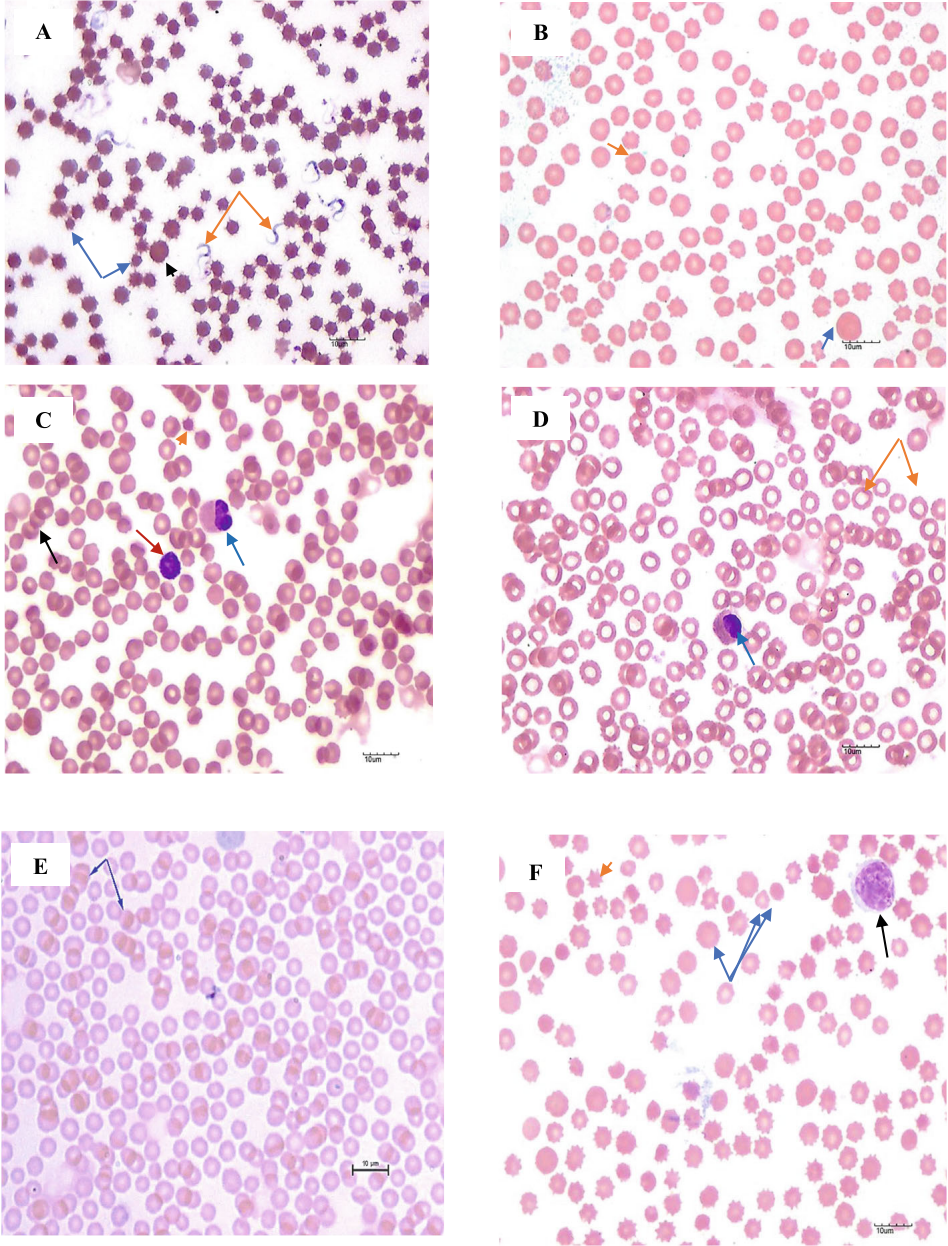
Micrographs of stained-blood smears from selected blood pathogen species infected Kedah-Kelantan x Brahman cattle groups from Muadzam Pahang Malaysia (× 1000 magnification). Confirmation of the types of blood pathogens were based on PCR and sequence analysis of the PCR amplicons **(A)** Double-orange arrow shows *Trypanosoma evansi* in a Wright-stained thin blood smear of Kedah-Kelantan X Brahman cattle. Anisocytosis with mixture of large (macrocytes), normal and small (spherocytes) erythrocytes are prominent; the presence of echinocytes of this blood smear are more related to drying effect. Double-blue arrow shows spherocytes while black arrow head shows a macrocyte **(B)** A Wright-stained thin blood smear from *Anaplasma platys + Theileria sinensis* double species co-infected cattle showing the presence of echinocytes (orange arrow represents one of the ecchinocytes) and a macrocyte (blue arrow). Poikilocytosis and anisocytosis are evident. **(C)** A Wright-stained thin blood smear from *Anaplasma marginale + T. orientalis* double species co-infected cattle showing spherocyte (orange-arrow head)metamyelocyte (blue arrow), and lymphocyte (red arrow) with background erythrocytes of different sizes (anisocytosis), and rouleaux formation (black arrow). **(D)** Wright-stained thin blood smears from *Theileria orientalis* + *T. sinensis* double species co-infected cattle showing numerous erythrocytes with wide central pallor (hypochromasia) and different sizes (anisocytosis). Blue arrow shows a metamyelocyte **(E)** Wright-stained thin blood smear of *C. M. haemobos + T. sinensis* double species co-infected cattle showing marked rouleaux formation with normal erythrocyte size. **(F)** Thin blood smear from *C. M. haemobos* + *T. sinensis* + *A. marginale* triple species co-infected cattle showing poikilocytosis and anisocytosis (represented by triple blue arrows). Large lymphocyte (black arrow) and erythrocytes of varying sizes [(macrocytes, normal and spherocytes (triple blue arrows)], poikilocytosis and hypochromasia are evident. Orange arrowhead shows an echinocyte. Note: Macrocytes reflect the regenerative response of anaemia, spherocytes reflect an immune-mediated haemolytic anaemia (IMHA) state, metamyelocytes (indicates moderate left shift) and rouleaux formation reflect a systemic inflammatory response in the cattle

Clinical signs such as anorexia, profuse ocular discharge, emaciation, weakness, and dyspnoea were observed in most of the sampled animals. Only two cattle were recumbent and had very poor body condition, cachexic and with less detectable body fat. Tail head, spine and ribs were prominent. The outline of spine and ribs of all other clinically ill cattle were slightly visible. Pallor of mucous membrane was observed in fifteen (15/61, 24.6%) animals. No *Theileria* piroplasms or schizonts were detected in the blood cells. *Anaplasma* morula were absent in the erythrocytes and *C. M. haemobos* were not seen attached onto the surface of erythrocytes. Numerous *T. evansi* were seen in the thin blood smear as elongated extracellular protozoan parasites (Fig. 1A). The blood picture of all cattle group revealed the presence of echinocytes, elliptocytes, macrocytes, spherocytes, anisocytosis, few acanthocytes, rouleaux formation and immature neutrophils (metamyelocytes) (Figs. 1A-F).

### Molecular identification, detection rate and similarity analysis of the blood pathogen strains detected in the KK x Brahman cattle

**Fig. 2:**
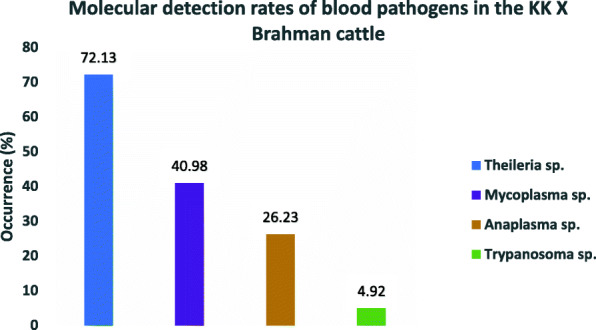
Molecular detection rates of *Theileria*, *Mycoplasma*, *Anaplasma* and *Trypanosoma species* confirmed by PCR amplification and sequencing in the Kedah-Kelantan x Brahman cattle sampled in Muadzam Pahang Malaysia

Molecular analysis by PCR amplification using genus specific primers produced expected fragment sizes of approximately 1750 bp for 18S rRNA gene of *Theileria* species, 345 bp for 16SrRNA gene of *Anaplasma* species and 280 bp for 16SrRNA gene of *Mycoplasma* species. *Theileria* species were detected in 44/61 cattle (72.13%) and was the blood pathogen with the highest molecular detection rate, followed by *Mycoplasma* species in 25/61 (40.98%) cattle, *Anaplasma* species in 16/61 cattle (26.23%) and *Trypanosoma evansi* in 3/61 cattle (4.92%) (Fig. 2). To identify and further confirm the specific species of blood pathogens detected in the cattle, PCR amplification using species-specific primers produced fragment of approximately 776 bp of the major piroplasm surface protein (MPSP) gene of *T. orientalis* in 20/61 cattle (32.79%)*,* 761 bp of major surface protein 4 (MSP4) gene of *A. marginale* in 13/61 cattle (21.31%), 279 bp of 16SrRNA gene of *C. M. haemobos* in 25/61 cattle (40.98%) and 205 bp of RoTat1.2 VSG gene of *T. evansi* in 3/61cattle (4.92%). Sequencing of *T. orientalis* PCR amplicons yielded 455–720 protein-coding nucleotides (MT184275-MT184279) and demonstrated between 98.17–99.80% molecular similarity with reference sequences from Thailand (AB562570.1), Vietnam (LC125432.1), Sri Lanka (LC438477.1; AB701444.1), Myanmar (AB871365.1), China (KU356867.1). Twenty-four out of fourty-four (44) *Theileria* species PCR amplicons, which were negative for *T. orientalis* were amplified using the 18S rRNA gene primer sets. Fourteen out of 24 amplicons were randomly selected for sequencing and yielded between 1651 and 1698 nucleotides. Similarity analysis using nucleotide BLAST (BLASTn) demonstrated that those fourteen (14) negative *T. orientalis* PCR amplicons corresponded to *T. sinensis* (MT271898 - MT271911), and shared a high degree of molecular similarity (99.5–99.87%) in comparison with reference sequences from China (KF559355.1; EU274472.1; HM538203.1), 99.7% similarity with reference strain from Johore Malaysia (KX263953.1) and 99.65% similarity with Thailand strain (MH156036.1).

The sixteen (16) positive 16S ribosomal RNA gene of *Anaplasma* species strains further subjected to a species-specific PCR using MSP4 primer set, revealed that 13 out of 16 amplicons were positive for *A. marginale.* Five representative amplicon sequences (MT173810 - MT173814) for MSP4 gene of *A. marginale* yielded 634–704 protein-coding nucleotides and demonstrated 99–100% similarity for *A. marginale* reference sequences from Western Cuba (MK809386), Brazil (KM624516), Thailand (MK140740) and India (KX989516). The 2 out of 3 amplicons negative for *A. marginale* were sequenced using 16S rRNA primer set and demonstrated 99% similarity for *A. platys* reference sequences from China (KU586159.1) and Brazil (JX392984.1), and 98% similarity with *A. platys* strains from India (MG050139.1). The *A. platys* strains MT259465 & MT259466 from this study had 98.03% similarity with reference *A. platys* strain (MH686048.1) detected in two Vietnamese cattle [23]. A 99.24% molecular similarity existed between *A. platys* strains from this study and other *A. platys* strains from stray dogs in several Malaysian states; Sarawak (KU500914.1), Selangor (KU500912.1), Sabah (KU500909.1), Pahang (KU500906.1), Pinang (KU500905.1), Kedah (KU500904.1) and Johor (KU500902.1). *A. platys* strains from this study also demonstrated 100% similarity with *A. platys* Malaysian strain (MH368782) adapted from Koh et al. [57].

16SrRNA gene sequences of *C. M. haemobos* strains (MT521752 - MT521753) from this study shared 100% molecular similarity with reference strains from Malaysia (KT985638.1), Switzerland (EF616468), Cuba (MG948633.1), Brazil (JN314393.1), China (MH388479.1), Mozambique (MF992084.1), Philippines (MK328955.1) and 99.57% similarity with reference sequence from Japan (EU367965.1). Amplicon sequences for RoTat1.2 VSG gene of *T. evansi* strains from this study (MT514513-MT514514) demonstrated 100% molecular similarity with strains from Kenya (MK867832; MK867833), India (KY457409.1) and Egypt (KF726106; JX888091). No significant similarity was found between our strains and local Malaysian *T. evansi* strains (JN247439.1; AM497933.1; AM497934.1).

### Percentage occurrence of single, double and triple species co-infection in Kedah-Kelantan x Brahman cattle

A total of six species of blood pathogens were detected by PCR and sequencing of amplicons and these include *T. orientalis*, *T. sinensis*, *A. marginale*, *A. platys*, *C. M. haemobos* and *T. evansi*. Detection rate of single and mixed blood pathogen infection are presented in Table 3. Analysis of blood pathogen single species infection among the sampled cattle revealed that 14.75% were infected by *T. sinensis* while sole infections with *C. M. haemobos* infected cattle and *T. evansi* were 8.20 and 4.92% respectively. *Theileria sinensis + C. M. haemobos* had the highest double species co-infection (27.87%), followed by *T. orientalis + A. marginale* (16.39%), *T. orientalis* + *T. sinensis* (14.75%), and the lowest being *A. platys* + *T. sinensis* (4.92%) (Table 3). *C. Mycoplasma haemobos* + *T. sinensis* + *A. marginale* triple species co-infection was detected in (8.20%) of the sampled cattle. *T. sinensis* + *C. M. haemobos* species co-infection had the highest occurrence while *C.M. haemobos* + *T. sinensis* + *A. marginale* triple species co-infection was the least (Table 3). Forty-four cattle (72.13%) were infected with more than one blood pathogen. There were no significant association between molecular presence of the blood pathogens and age (χ2 = 23.001; *p* = 0.060) or blood pathogens and gender (χ2 = 9.524; *p* = 0.217).

**Table 3:** Percentage occurrence of single and mixed blood pathogen infections in Kedah-Kelantan x Brahman cattle

Blood Pathogens	No of cattle positive for blood pathogens	Occurrence (%) (95% CI)
*Trypanosoma evansi*	3	4.92 (1.69–13.49)
*C. Mycoplasma haemobos*	5	8.20 (3.55–17.79)
*Theileria sinensis*	9	14.75 (7.96–25.72)
*Theileria orientalis + Anaplasma marginale*	10	16.39 (9.16–27.61)
*Anaplasma platys + Theileria sinensis*	3	4.92 (1.69–13.49)
*Theileria sinensis + C. Mycoplasma haemobos*	17	27.87 (18.19–40.17)
*Theileria orientalis + Theileria sinensis*	9	14.75 (7.96–25.72)
*C. M. haemobos + T. sinensis + A. marginale*	5	8.20 (3.55–17.79)

*CI* Confidence Interval. Total number of clinically ill cattle = 61. Total number of blood pathogen positive cattle = 61. Total number of cattle with more than one blood pathogen infection = 44

### Phylogenetic analysis

**Fig. 3:**
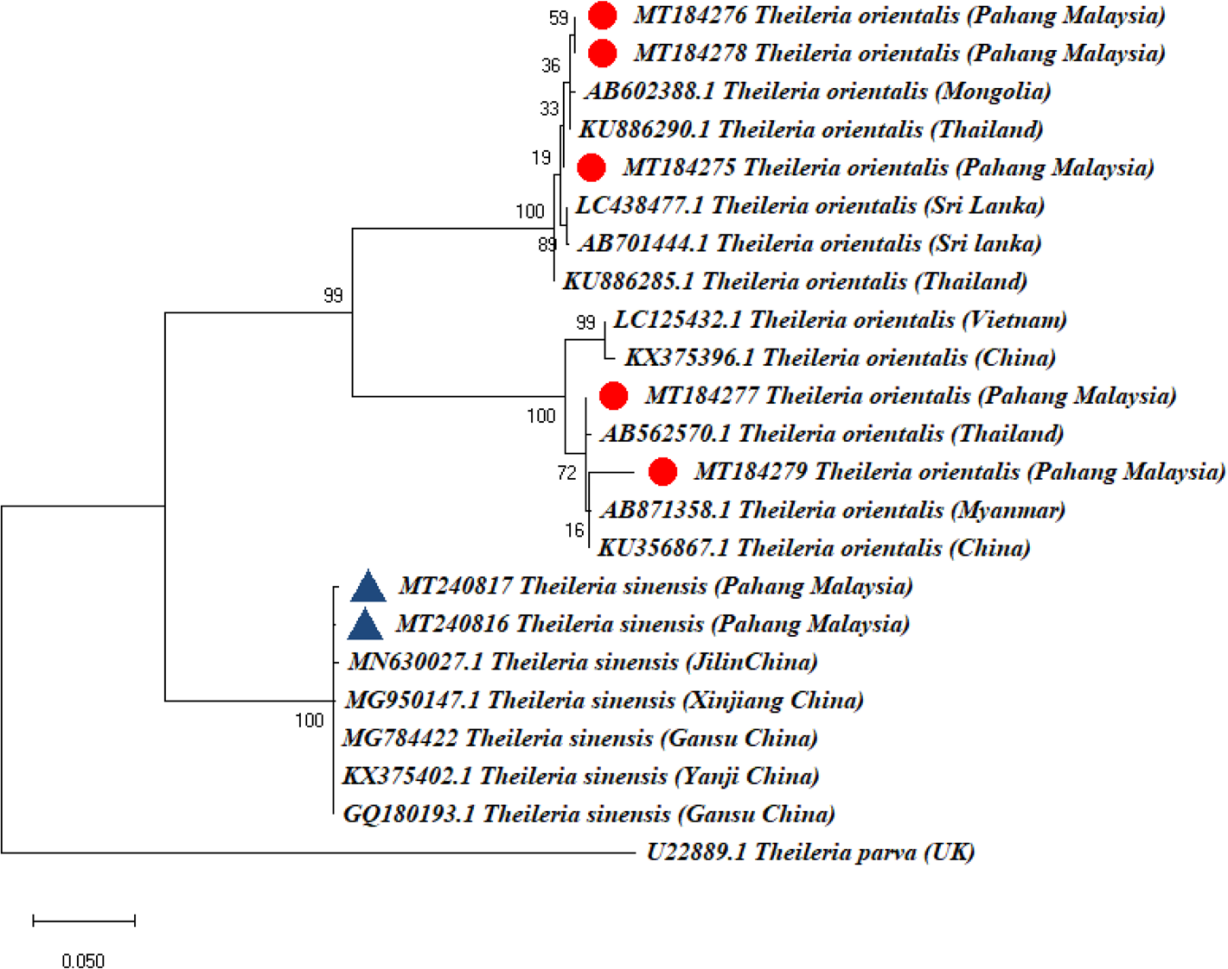
Phylogenetic tree showing taxonomic relationships of *Theileria orientalis* and *Theileria sinensis* detected from Kedah Kelantan x Brahman cattle in Muadzam Pahang Malaysia. The phylogenetic relationships were inferred based on MPSP gene (455–720 bp) from five (5) sequences of *T. orientalis* and two (2) MPSP gene sequences of *T. sinensis* (871–872 bp)*. Theileria parva* was used as an outgroup. Phylogenetic reconstruction was performed in MEGA X using maximum likelihood analysis based on the general time-reversible model. Support for each node was indicated by 1000 bootstrap resamplings. The scale bar represents the number of substitutions per site. The accession numbers are given at beginning of each sequence label, followed by the full species names and country, in parenthesis, from where they were obtained. Five (5) *T. orientalis* and two (2) *T. sinensis* strains from Muadzam Pahang Malaysia are marked with a solid red circle and blue triangles respectively

Phylogenetic analysis was done to determine whether the vector-borne blood pathogens detected in this study are genetically diverse within different geographical regions of the world. Phylogenetic analysis based on the MPSP gene of *T. orientalis* grouped our Malaysian strains, MT184277 and MT184279 together with reference strains from China (KX375396.1; KU356867.1), Vietnam (LC125432.1), Thailand (AB562570) and Myanmar (AB871365.1) in a subclade. Thailand (KU886290.1; KU886285.1), Sri Lanka (AB701444; LC438477.1) and Mongolia (AB602388.1) reference strains grouped together and formed a subclade with our Malaysian strains MT184275, MT184276 and MT184278 (Fig. 3). The MPSP gene sequences of *T. sinensis* (MT250816–7) from this study grouped together with sequences from Jilin China (MN630027.1), Xinjiang China (MG950147.1), Gansu China (MG784422; GQ180193.1) and Yanji China (KX375402.1) (Fig. 3) but formed a separate branch with our five *T. orientalis* MPSP gene sequences (MT259465-MT259466),.

**Fig. 4:**
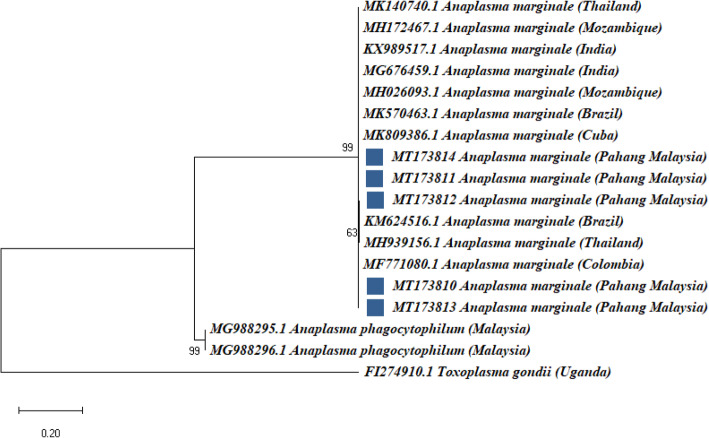
Maximum Likelihood analysis of MSP4 gene of *A. marginale* sequences showing the phylogenetic relationship of the *Anaplasma* species. The phylogenetic relationship was inferred based on MSP4 gene (634–704 bp) from five (5) sequences of *A. marginale* strains, ten MSP4 reference gene sequences of *A. marginale* and two A. phagocytophilum MSP4 gene sequences. *Toxoplasma gondii* was used as an outgroup. The scale bar represents the number of substitutions per site. For each sequence, the accession numbers are given at beginning of each sequence label, the full species names are followed up by the country in parenthesis where they were obtained. Five *A. marginale* strains detected in Kedah-Kelantan x Brahman cross bred cattle in Pahang Malaysia are marked with a solid blue square

Phylogenetic analysis based on MSP4 gene grouped *A. marginale* strains (MT173810-MT173814) from this study together with reference sequences from Mozambique (MH172467.1; MH026093.1), Colombia (MF771080.1), Thailand (MK140740.1), India (KX989517; MG676459.1), Brazil (MK570463.1) and Cuba (MK809386.1) (Fig. 4). Five *A. marginale* MSP4 gene sequences from this study formed separate branches with *A. phagocytophilim* Malaysian strains (MG988295.1-MG988296.1) (Fig. 4).

**Fig. 5:**
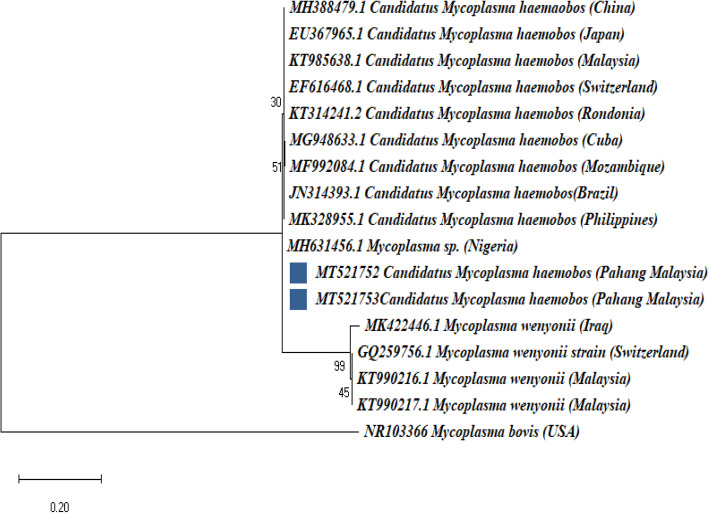
Phylogenetic tree showing taxonomic relationships of *Candidatus Mycoplasma haemobos* detected from KK x Brahman cattle in Pahang Malaysia. The relationships were inferred based on 16SrRNA gene sequences from two (2) *C. M. haemobos* (248 bp & 253 bp), ten (10) reference sequences of *C. Mycoplasma haemobos* and four reference sequences of *Mycoplasma wenyonii*. *Mycoplasma bovis* was used as an outgroup. Phylogenetic reconstruction was performed in MEGA X using maximum likelihood analysis based on the general time-reversible model. Support for each node was indicated by 1000 bootstrap resamplings. The scale bar represents the number of substitutions per site. For each sequence, the full species names are followed up by the country where they were isolated. The accession numbers are given at beginning of each sequence label. Two *C. Mycoplasma haemobos* strains from Pahang Malaysia are marked with a solid blue square

**Fig. 6:**
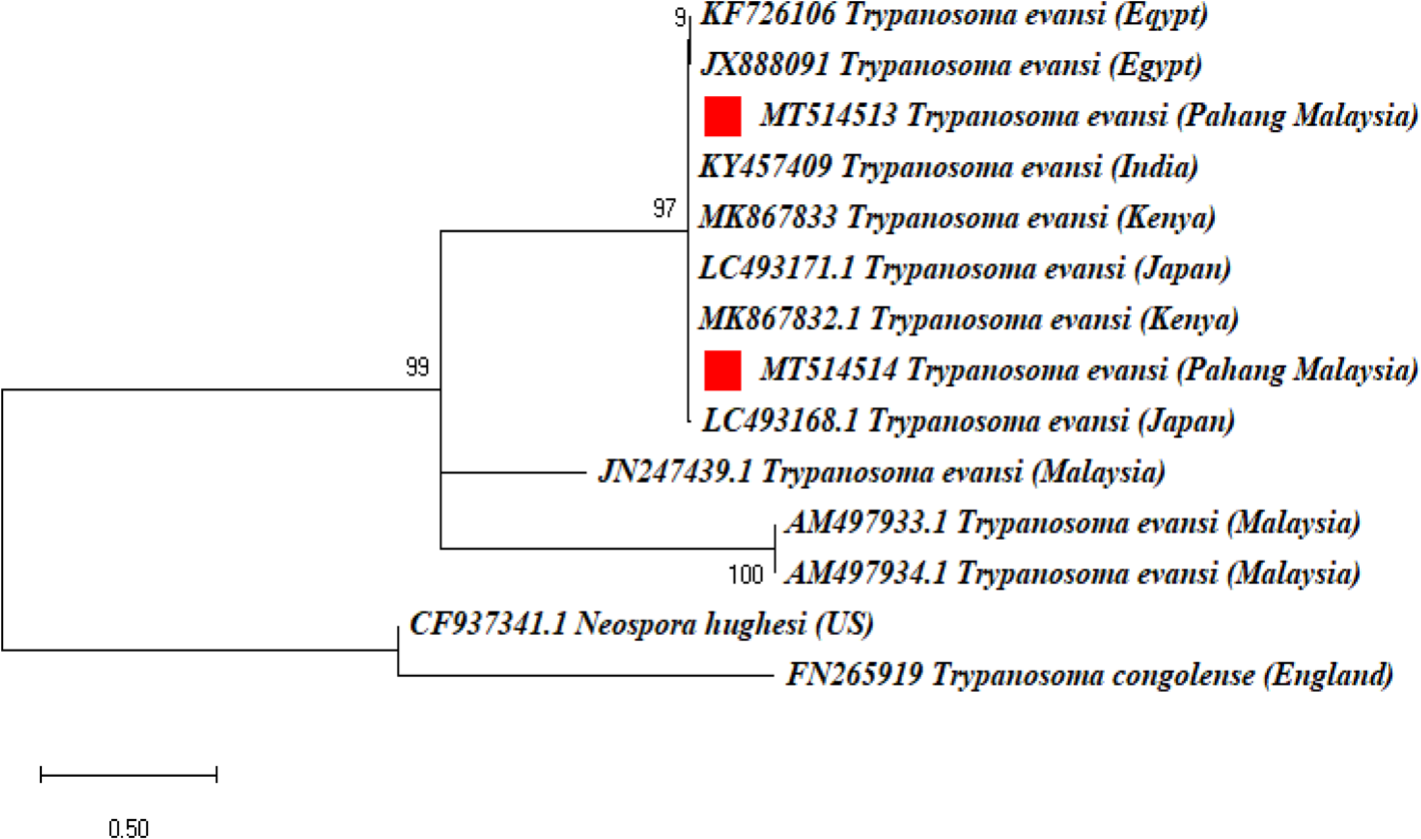
Phylogenetic tree showing taxonomic relationships of *Trypanosoma evansi* detected from KK x Brahman cattle in Muadzam Pahang Malaysia. The relationships were inferred based on RoTat1.2 VSG gene (203–204 bp) from two *T. evansi* from Pahang Malaysia and 10 reference sequences of *T. evansi*. *Neospora hughesi* and *Trypanosoma congolense* were used as outgroup. Phylogenetic reconstruction was performed in MEGA X using maximum likelihood analysis based on the general time-reversible model. Support for each node was indicated by 1000 bootstrap resamplings. The scale bar represents the number of substitutions per site. For each sequence, the full species names are followed up by the country where they were isolated. The accession numbers are given at beginning of each sequence label. Two (2) *Trypanosoma evansi* strains detected in the cattle from Muadzam Pahang Malaysia are marked with a solid red square

16S rRNA reference gene sequences of *C. M. haemobos* from China (MH388479.1), Japan (EU367965.1), Malaysia (KT985638.1), Switzerland (EF616468.1), Rondonia (KT314241.1), Cuba (MG948633.1), Mozambique (MF992084.1), Brazil (JN314393.1), Philippines (MK328955.1) and Nigeria (MH631456.1) were tightly grouped together with *C. M. haemobos* strains from this study (MT521752 – MT521753). *Mycoplasma wenyonii* strains from Iraq (MK422446.1), Switzerland (GQ259756.1) and Malaysia (KT990216-KT990217.1) formed a separate branch from the *C. M. haemobos* strains from this study (Fig. 5). Phylogenetic analysis revealed that the 2 *Trypanosoma evansi* RoTat1.2 VSG sequences from this study (MT514513 and MT514514) grouped together with reference strains from Egypt (KF726106.1; JX888091.1), India (KY457409), Kenya (MK867832.1-MK867833.1) and Japan (LC493168.1; LC493171.1). Surprisingly, the three Malaysian reference *T. evansi* strains (JN247439.1; AM497933.1-AM497934.1) formed a separate branch from the *T. evansi* strains obtained from this study (Fig. 6).

### Haemato-biochemical and electrolyte abnormalities in the different cattle groups

The haematology and serum biochemistry profile of sampled KK x Brahman crossbred cattle are presented in Tables 4 and 5. Anaemia was detected in 15/61 cattle (24.59, 95% CI 15.51–36.68%) and icterus in 3/61 cattle (4.92, 95% CI 1.69–13.49%). The cattle were divided into eight groups namely *T. evansi* infected cattle; *C. M. haemobos* infected cattle; *T. sinensis* infected cattle; *T. orientalis + A. marginale* infected cattle; *A. platys + T. sinensis* infected cattle; *T. sinensis + C. M. haemobos; T. orientalis + T. sinensis* infected cattle; *C. M. haemobos + T. sinensis + A. marginale* infected cattle groups. A significant (*p* < 0.05) decrease in mean PCV, RBC, Hb and high mean cell volume (MCV), icteric index and a normal MCHC value were recorded for the *T. evansi* infected group. Plasma protein value was significantly (*p* < 0.05) elevated in all cattle groups with that of the *T. evansi* being highest when compared to the clinically healthy cattle. Cattle positive for *T. sinensis* sole infection had significantly (*p <* 0.05) low mean RBC, Hb, PCV values and normal icteric index. Significantly (*p <* 0.05) decreased mean RBC count and Hb values with a high MCV and normal PCV values were recorded in the *A. platys + T. sinensis* cattle group. Significant (*P* < 0.05) increase in mean plasma protein values were recorded in all infected cattle groups when compared to clinically healthy cattle. There were no significant (*p* > 0.05) differences in the mean values of MCHC and platelet count of all cattle groups. All blood pathogen infected cattle groups had significantly (*p* < 0.05) high mean myelocyte, metamyelocyte, band and segmented neutrophil values when compared to the clinically healthy cattle (Table 4). Significant (*p <* 0.05) increase in mean values of sodium, potassium and chloride were recorded across all infected cattle groups, with a significant increase in mean creatinine and urea values recorded only for the *T. evansi* infected cattle group (Table 5). Significant (*p <* 0.05) increases in mean serum ALP, AST, GGT activities, globulin, total and conjugated bilirubin, and decreased mean serum albumin value were observed in the *T. evansi* cattle group when compared to the other infected groups and clinically healthy cattle group (Table 5). The mean serum alkaline phosphatase (ALP) value of the *T. sinensis* infected cattle was lower than that of all the cattle groups. Mean serum total protein and globulin values were increased in the *T. evansi*, *C. M. haemobos* and *T. orientalis + A. marginale* infected cattle groups. Low albumin with low albumin to globulin ratio (A:G) was recorded in the *T. evansi* group, while albumin to globulin ratio (A:G) was decreased across all cattle groups. Significant (*p* < 0.05) increases in the mean serum total and unconjugated bilirubin values of the *T. evansi* cattle were recorded when compared to the other cattle groups (Table 5).

**Table 4:** Haematological parameters of KK x Brahman cattle naturally infected by blood pathogens

Haematological Parameters	***T. evansi*** infected cattle (***n =*** 3)	***Candidatus M. haemobos*** infected cattle (***n =*** 5)	***T. sinensis*** infected cattle (***n =*** 9)	***T. orientalis + A. marginale*** infected cattle (***n =*** 10)	***A. platys + T. sinensis*** infected cattle (***n =*** 3)	***T. sinensis + C. M. haemobos*** infected cattle (***n*** = 17)	***T. orientalis + T. sinensis*** infected cattle (***n*** = 9)	***C. M. haemobos + T. sinensis + A. marginale*** infected cattle (***n*** = 5)	Clinically healthy cattle (***n*** = 17)
**PCV (L/L)**	0.20 ± 0.01^a^	0.32 ± 0.04^c^	0.23 ± 0.01^ab^	0.33 ± 0.02^cd^	0.32 ± 0.06^c^	0.33 ± 0.01^cd^	0.34 ± 0.02^cd^	0.30 ± 0.02^bc^	0.40 ± 0.01^d^
**RBC count (× 10**^**12**^**/L)**	3.03 ± 0.03^a^	6.47 ± 0.38^bcd^	3.86 ± 0.37^a^	5.83 ± 0.45^bc^	4.78 ± 0.45^ab^	7.05 ± 0.41^cd^	6.47 ± 0.52^bcd^	6.11 ± 0.51^bcd^	8.05 ± 0.23^d^
**Haemoglobin (g/L)**	62.50 ± 0.50^a^	84.33 ± 0.50^bcd^	67.88 ± 2.82^ab^	95.63 ± 4.84^cd^	78.00 ± 0.00^abc^	103.00 ± 3.78^d^	87.00 ± 5.99^bcd^	86.00 ± 2.35^bc^	126.14 ± 2.02^e^
**MCV (fL)**	66.09 ± 2.76^bc^	48.68 ± 5.54^a^	54.56 ± 3.28^ab^	58.63 ± 3.43^abc^	68.79 ± 3.14^c^	49.75 ± 2.53^a^	54.77 ± 3.83^ab^	54.23 ± 7.01^ab^	49.31 ± 1.02^a^
**MCHC (g/L)**	328.00 ± 4.00	339.75 ± 6.32	293.03 ± 5.79	329.67 ± 4.26	321.00 ± 3.00	330.83 ± 5.75	329.78 ± 4.26	330.83 ± 5.76	311.53 ± 1.11
**Plasma proteins (g/L)**	109.00 ± 1.41^c^	86.25 ± 5.07^ab^	91.11 ± 2.65^ab^	93.60 ± 4.11^b^	95.00 ± 5.00^b^	95.35 ± 3.10^b^	90.63 ± 4.12^ab^	89.50 ± 4.11^ab^	77.13 ± 1.15^a^
**Icteric index (units)**	50.00 ± 0.00^b^	5.00 ± 0.00^a^	5.00 ± 0.00^a^	5.00 ± 0.00^a^	5.00 ± 0.00^a^	5.00 ± 0.00^a^	5.00 ± 0.00^a^	5.00 ± 0.00^a^	5.00 ± 0.00^a^
**Platelets (× 10**^**9**^**/L)**	321.50 ± 6.50^a^	392.00 ± 2.36^a^	378.11 ± 7.42^a^	357.44 ± 5.01^a^	420.00 ± 7.00 ^ab^	458.72 ± 6.59^ab^	322.25 ± 9.89^a^	600.33 ± 8.61^b^	461.51 ± 3.35^ab^
**WBC count (× 10**^**9**^**/L)**	4.94 ± 0.28	5.22 ± 0.65	5.07 ± 0.58	4.97 ± 0.93	6.96 ± 0.72	6.75 ± 0.59	6.91 ± 0.54	6.36 ± 0.97	7.30 ± 0.30
**Lymphocyte (× 10**^**9**^**/L)**	3.97 ± 0.42	4.08 ± 0.71	3.81 ± 0.50	3.66 ± 0.52	5.47 ± 0.67	5.88 ± 0.52	5.24 ± 0.56	4.98 ± 0.82	2.5–7.5
**Promyelocyte (× 10**^**9**^**/L)**	0.00 ± 0.00^a^	0.00 ± 0.00^a^	0.01 ± 0.00^ab^	0.00 ± 0.00^a^	0.03 ± 0.01^b^	0.01 ± 0.00^ab^	0.00 ± 0.00^a^	0.00 ± 0.00^a^	0.00 ± 0.00^a^
**Myelocyte (× 10**^**9**^**/L)**	0.12 ± 0.07^a^	0.15 ± 0.09^ab^	0.20 ± 0.06^ab^	0.40 ± 0.18^ab^	0.16 ± 0.02^ab^	0.14 ± 0.04^ab^	0.34 ± 0.13^ab^	0.08 ± 0.05^ab^	0.00 ± 0.00^a^
**Metamyelocyte (× 10**^**9**^**/L)**	0.40 ± 0.03^d^	0.22 ± 0.08^bc^	0.26 ± 0.10^bcd^	0.20 ± 0.04^bc^	0.29 ± 0.02^cd^	0.12 ± 0.03^abc^	0.16 ± 0.04^abc^	0.15 ± 0.08^abc^	0.00 ± 0.00^a^
**Band (× 10**^**9**^**/L)**	0.27 ± 0.11^abc^	0.66 ± 0.06^a^	0.34 ± 0.08^bc^	0.39 ± 0.11^bc^	0.25 ± 0.06^abc^	0.29 ± 0.05^abc^	0.52 ± 0.14^c^	0.85 ± 0.07^c^	0.00 ± 0.00^a^
**Segmented Neutrophil (× 10**^**9**^**/L)**	0.18 ± 0.04^a^	0.11 ± 0.02	0.11 ± 0.02^a^	0.20 ± 0.04^a^	0.27 ± 0.16^a^	0.31 ± 0.04^a^	0.31 ± 0.08^a^	0.29 ± 0.11^a^	3.06 ± 0.15^b^
**Eosinophil (× 10**^**9**^**/L)**	0.03 ± 0.01^a^	0.31 ± 0.17^ab^	0.27 ± 0.11^ab^	0.78 ± 0.04^b^	0.33 ± 0.21^ab^	0.50 ± 0.05^ab^	0.49 ± 0.03^ab^	0.70 ± 0.16^b^	0.52 ± 0.07^ab^
**Basophil (× 10**^**9**^**/L)**	0.00 ± 0.00^a^	0.05 ± 0.02^c^	0.06 ± 0.01^c^	0.01 ± 0.01^ab^	0.00 ± 0.00^a^	0.00 ± 0.00^a^	0.00 ± 0.00^a^	0.04 ± 0.02^bc^	0.00 ± 0.00^a^
**Monocyte (× 10**^**9**^**/L)**	0.10 ± 0.01^a^	0.08 ± 0.03^a^	0.44 ± 0.11^a^	0.12 ± 0.04^a^	0.11 ± 0.05^a^	0.20 ± 0.05^a^	0.16 ± 0.33^a^	0.17 ± 0.03^a^	0.46 ± 0.05^a^

^**a, b, ab, c, bc, cd, abc, bcd**^**Different superscripts indicate significant difference across the different blood pathogen infected cattle groups,** ***p*** **< 0.05**

**Table 5:** Serum biochemistry parameters of Kedah-Kelantan X Brahman cattle naturally infected by blood pathogens

Serum biochemical parameters	***T. evansi*** infected cattle (***n =*** 3)	***Candidatus M. haemobos*** infected cattle (***n =*** 5)	***T. sinensis*** infected cattle (***n =*** 9)	***T. orientalis*** + ***A. marginale*** infected cattle (***n =*** 10)	***A. platys*** + ***T. sinensis*** infected cattle (***n =*** 3)	***T. sinensis + C. M. haemobos*** infected cattle (***n =*** 17)	***T. orientalis + T. sinensis*** infected cattle (***n =*** 9)	***C. M. haemobos + T. sinensis + A. marginale*** infected cattle (***n =*** 5)	Clinically healthy cattle (***n =*** 17)
**ALP (U/L)**	351.00 ± 5.00^d^	54.00 ± 6.35^ab^	44.67 ± 4.22^a^	57.50 ± 6.97^ab^	51.00 ± 5.14^ab^	82.35 ± 5.53^bc^	65.63 ± 6.26^ab^	107.25 ± 6.07^c^	71.94 ± 4.62^bc^
**AST (U/L)**	135.50 ± 2.50^a^	57.50 ± 5.92^b^	81.00 ± 9.08^b^	68.10 ± 9.17^b^	67.00 ± 6.00^b^	70.13 ± 5.53^b^	71.13 ± 5.18^b^	62.40 ± 7.69^b^	78.86 ± 2.48^b^
**γ-GT (U/L)**	58.00 ± 1.00^b^	19.50 ± 3.07^a^	17.11 ± 2.90^a^	31.20 ± 6.06^a^	25.50 ± 6.50^a^	20.38 ± 2.95^a^	22.11 ± 4.28^a^	29.20 ± 3.71^a^	22.79 ± 1.48^a^
**Total proteins (g/L)**	92.40 ± 1.80^c^	76.93 ± 4.05^a^	75.29 ± 2.95^ab^	79.73 ± 2.58^b^	76.40 ± 1.00^ab^	76.17 ± 1.68^ab^	78.06 ± 2.80^b^	76.13 ± 2.06^ab^	71.85 ± 2.04^ab^
**Albumin (g/L)**	21.75 ± 0.65^a^	27.95 ± 1.35^b^	30.46 ± 0.99^bc^	31.52 ± 0.80^bc^	32.90 ± 3.40^bcd^	32.62 ± 0.82^bcd^	33.67 ± 1.19^cd^	32.30 ± 2.03^bc^	37.29 ± 0.75^d^
**Globulin (g/L)**	70.65 ± 1.15^b^	48.98 ± 2.71^a^	44.83 ± 2.57^a^	48.21 ± 2.11^a^	43.50 ± 2.40^a^	43.56 ± 1.54^a^	44.39 ± 2.42^a^	43.83 ± 1.57^a^	37.84 ± 1.03^a^
**A: G (Unit)**	0.31 ± 0.01^a^	0.72 ± 0.02^b^	0.70 ± 0.04^b^	0.67 ± 0.03^b^	0.84 ± 0.14^bc^	0.75 ± 0.21^b^	0.73 ± 0.04^b^	0.76 ± 0.07^b^	1.01 ± 0.03^c^
**Total bilirubin (**μmol**/L)**	30.30 ± 0.50^c^	5.05 ± 0.93^ab^	9.27 ± 1.57^b^	8.03 ± 1.29^ab^	5.30 ± 0.01^ab^	9.03 ± 1.04^b^	7.37 ± 0.81^ab^	7.88 ± 1.51^ab^	3.87 ± 0.30^a^
**Conjugated bilirubin (μmol/L)**	8.50 ± 0.20^b^	2.23 ± 0.44^a^	3.58 ± 0.54^a^	3.40 ± 0.47^a^	2.30 ± 0.30^a^	3.17 ± 0.27^a^	2.75 ± 0.36^a^	3.18 ± 0.51^a^	2.17 ± 0.13^a^
**Unconjugated bilirubin (μmol/L)**	21.80 ± 0.30^e^	2.83 ± 0.53^ab^	5.69 ± 1.05^cd^	4.63 ± 0.85^bcd^	3.00 ± 0.30^abc^	4.83 ± 0.50^bcd^	4.61 ± 0.48^bcd^	4.70 ± 1.03^bcd^	1.41 ± 0.21^a^
**Sodium (mmol/L)**	191.50 ± 6.50^b^	209.00 ± 6.06^b^	187.22 ± 7.22^b^	187.56 ± 6.20^b^	168.50 ± 7.19^ab^	180.78 ± 8.72^ab^	179.89 ± 6.93^ab^	171.50 ± 3.00^ab^	141.00 ± 1.87^a^
**Potassium (mmol/L)**	6.15 ± 0.05^b^	6.50 ± 0.58^b^	5.86 ± 0.19^ab^	6.10 ± 0.47^b^	5.20 ± 1.20^ab^	5.76 ± 0.24^ab^	5.57 ± 0.29^ab^	5.28 ± 0.39^ab^	4.65 ± 0.10
**Chloride (mmol/L)**	126.50 ± 5.50^ab^	150.50 ± 4.45^b^	133.33 ± 6.15^ab^	129.40 ± 5.74^ab^	117.50 ± 3.32^a^	126.72 ± 6.70^ab^	126.44 ± 5.10^ab^	118.00 ± 9.58^a^	103.00 ± 1.01^a^
**Inorganic phosphate (mmol/L)**	2.75 ± 0.05	2.35 ± 0.40	2.38 ± 0.17	2.54 ± 0.12	2.65 ± 0.65	2.82 ± 0.15	2.73 ± 0.15	2.68 ± 0.27	2.10 ± 0.07
**Creatinine (μmol/L)**	182.00 ± 1.00^d^	104.75 ± 6.87^b^	85.00 ± 4.17^a^	104.30 ± 3.60^ab^	95.00 ± 2.00^ab^	113.59 ± 3.25^b^	104.57 ± 4.11^ab^	112.75 ± 4.25^b^	136.74 ± 3.17^c^
**Urea (mmol/L)**	11.30 ± 1.00^d^	6.88 ± 1.11^abc^	6.11 ± 0.51^ab^	7.24 ± 0.54^abc^	8.40 ± 0.30^c^	7.94 ± 0.26^bc^	7.68 ± 0.54^bc^	7.24 ± 0.48^abc^	5.27 ± 0.24^a^

^**a, b, ab, c, bc, cd, abc, bcd**^**Different superscripts indicate significant difference across the different blood pathogen infected cattle groups,** ***p*** **< 0.05**

### Mean erythrocyte osmotic fragility

**Fig. 7:**
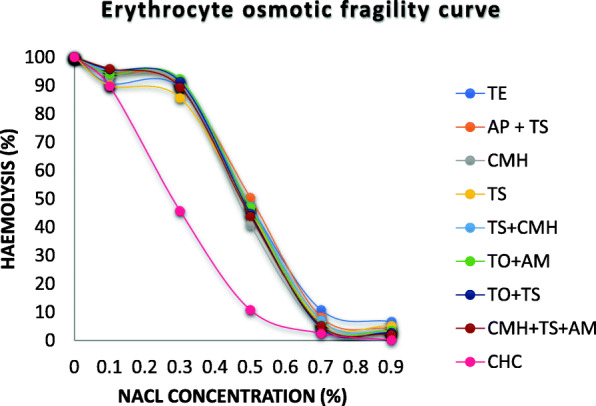
The erythrocyte osmotic fragility curves of the different blood pathogen infected Kedah-Kelantan X Brahman cattle groups from Muadzam Pahang Malaysia. Blood pathogen infection was confirmed by PCR amplification and sequencing. The erythrocyte osmotic fragility values were obtained by subjecting blood samples to different sodium chloride (NaCl) concentrations (0–0.9%). All curves showed a rightward trend which signifies high fragility of red blood cells, except for the clinically healthy cattle. TE: *Trypanosoma evansi* infected cattle; AP + TS: *Anaplasma platys* + *Theileria sinensis*; CMH: *Candidatus Mycoplasma haemobos* infected cattle; TS: *Theileria sinensis* infected cattle; TO+AM: *Theileria orientalis* + *A. marginale* infected cattle, TS + CMH: *Theileria sinensis + Candidatus Mycoplasma haemobos* infected cattle, TO + TS: *Theileria orientalis* + *Theileria sinensis* infected cattle*;* CMH + TS + AM: *Candidatus Mycoplasma haemobos* + *Theileria sinensis* + *Anaplasma marginale* infected cattle; CHC: Clinically healthy cattle

**Fig. 8:**
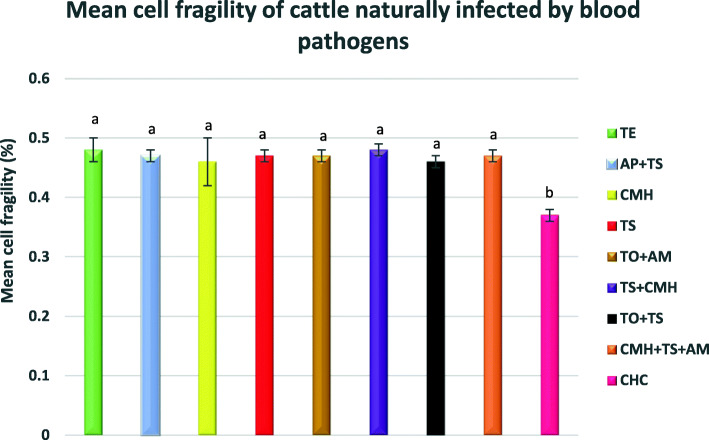
The mean cell fragility (MCF) values of different naturally infected Kedah-Kelantan x Brahman cattle groups from Muadzam Pahang Malaysia. The MCF values were extrapolated from the erythrocyte osmotic fragility curve in Fig. 7 and represents the saline concentration in which 50% haemolysis occurred. There was significant difference in the mean cell fragility of all blood pathogen infected cattle groups, relative to the clinically healthy cattle (*p* < 0.05). TE: *Trypanosoma evansi* infected cattle; AP + TS: *Anaplasma platys* + *Theileria sinensis*; CMH: *Candidatus Mycoplasma haemobos* infected cattle; TS: *Theileria sinensis* infected cattle; TO+AM: *Theileria orientalis* + *A. marginale* infected cattle, TS + CMH: *Theileria sinensis + Candidatus Mycoplasma haemobos* infected cattle, TO+TS: *Theileria orientalis* + *Theileria sinensis* infected cattle*;* CMH + TS + AM: *Candidatus Mycoplasma haemobos* + *Theileria sinensis* + *Anaplasma marginale* infected cattle. CHC: Clinically healthy cattle

The erythrocyte osmotic fragility (EOF) curves of all infected cattle groups showed an evident shift to the right, when compared to the clinically healthy cattle (Fig. 7). Duncan’s post hoc comparison test showed an increase of erythrocyte osmotic fragility at 0, 0.1, 0.3, 0.5, 0.7 and 0.9% saline concentration in all infected cattle groups (Fig. 7). The mean cell fragility (MCF) values of all the infected cattle groups were significantly (*p <* 0.05) higher than that of the clinically healthy cattle (Fig. 8).

## Discussion

Clinical signs observed in clinically ill cattle such as anaemia, anorexia, emaciation, profuse ocular discharges, loss of body condition, and recumbency are usually associated with blood pathogen infections in cattle [1–3, 17, 58]. In the present study, *T. orientalis, T. sinensis, A. marginale, A. platys, C. Mycoplasma haemobos*, and *T. evansi* were successfully amplified using a PCR-based technique. *Theileria* species were the predominant species detected in 44 out of 61 cattle. The high detection rate of *Theileria* DNA (72.13%) confirms that *Theileria* infection is endemic in a cattle farm in Pahang, with *T. sinensis* being the most commonly detected *Theileria* species. Molecular evidence of theileriosis being caused by *T. sinensis* and *T. orientalis* in Malaysia was first documented by Kho et al. [26] and Ola-Fadunsin et al. [59], respectively. However, the association between bovine anaemia and *T. sinensis* infection is presented for the first time in this study. *T. sinensis* is one of the major causative agents of bovine theileriosis in China, and it is transmitted by *Haemaphysalis qinghaiensis* ticks [32]. Therefore, its detection in Malaysian cattle suggests the possible introduction, importation, or close contact of an asymptomatic carrier cattle to naïve cattle in Malaysia. Consequently, *T. sinensis* is now indigenous to Malaysia and no longer restricted to China. In addition, the detection of *T. sinensis* in cattle ticks (*Rhipicephalus microplus* and *Haemaphysalis bispinosa*) found in Malaysia implies that these ticks potentially play important roles in the transmission of theileriosis [26]. *T. sinensis* was also recently detected in Russian cattle, which further indicates that the pathogen is no longer indigenous to Asia [60]. Phylogenetic analyses of the *Theileria*, *Anaplasma, C. Mycoplasma haemobos*, and *T. evansi* Malaysian strains showed that they are tightly grouped together and closely related to their respective reference strains from different geographic regions. The sequence similarity among the Malaysian strains and those in other parts of the world might have been caused by the movement/importation of cattle carrying the blood pathogens into Southeast Asia. Astonishingly, previously reported *T. evansi* Malaysian strains [61] are different from the strains reported in this study. This, however, proves that some degree of polymorphism exists among *T. evansi* strains detected in the same herd or in endemic areas (reviewed in Ref [62]). Besides *A. marginale,* the detection of another *Anaplasma* species (*A. platys*) in Malaysian cattle agrees with the findings of Koh et al. [57], who detected other *Anaplasma* species such as *A. capra, A. phagocytophilum*, and *Candidatus A. camelii* in domestic and wild animals in Peninsular Malaysia. *Anaplasma platys* is a rickettsial bacteria transmitted by *Rhipicephalus sanguineus* sensu lato that infects a wide range of hosts such as humans, horses, small ruminants, and preferentially dogs [21, 22]. It causes canine infectious cyclic thrombocytopenia, and its pathogenic role in humans, small ruminants, and horses is not fully understood but has been associated with chronic nonspecific clinical signs such as headaches and muscle pains in humans [63]. Humans in close contact with their pets usually become infected. Our discovery of *A. platys* in cattle agrees with the study by Chien et al. [23], which reported a similar finding for Vietnamese cattle. This pathogen has also been detected in cattle in Italy [64] and Nigeria [65]. The detection of *A. platys* in cattle raises an urgent concern about the zoonotic implication of this blood pathogen in Malaysia, as pastoralists, veterinarians, and para-veterinary personnel are at high risk of exposure. The high detection rates of blood pathogen co-infections recorded in this study is very common in cattle, and co-transmission of multiple pathogens enhances the disease severity and/or evolves with nonspecific clinical signs and symptoms, resulting in diagnostic complexities [66]. Co-infections with two to six different blood pathogens in cattle have been reported in Kenya [2], Malaysia [6], Russia [60], Uganda [67], Tanzania [68], and southwestern Ethiopia [69], and a majority of mixed infections usually occur as double infections [60, 69].

Significant changes in haemato-biochemical parameters were observed in the *T. evansi*-infected cattle group, *T. sinensis*-infected cattle group, and *A. platys + T. sinensis*-infected cattle group. Hemolytic anaemia in trypanosomosis is usually due to mechanical injury to the erythrocytes and to the subsequent lysis caused by the lashing action of Trypanosoma flagella and attachment of the trypanosomes onto red blood cell surfaces via sialic acid receptors [70]. The presence of spherocytes in the stained blood smears of *T. evansi-*infected cattle may be due to partial erythrophagocytosis of the autoantibodies and complement-coated erythrocytes [71]. Elevations in the serum total and unconjugated bilirubin in the *T. evansi* group were due to massive intravascular hemolysis with the increased release and breakdown of hemoglobin and the conversion to bilirubin by macrophages. Elevation of the liver enzyme activities in the *T. evansi* group was ascribed to cellular hypoxia and/or centrilobular necrosis due to a low liver blood volume, which leads to hepatocyte injury [72]. This hepatocellular injury could have contributed to the slow rate of bilirubin conjugation. Albumin is a negative acute phase protein that decreases in acute inflammatory conditions; therefore, the induction of inflammatory conditions by the trypanosomes could lead to hypoalbuminemia. The albumin-to-globulin ratio also decreased with an increase in inflammation. The toxic metabolites of the protozoa may have caused possible injury to the kidneys or renal insufficiency, hence the increased serum urea and creatinine levels in *T. evansi*-infected cattle [73]. In addition, prerenal dehydration could be responsible for the increase in serum urea and creatinine levels in *T. evansi-*infected cattle.

The cases of mild anaemia recorded in the *T. sinensis*-infected cattle group could be ascribed to extravascular hemolysis of abnormally shaped red blood cells performed by the mononuclear phagocyte system [74]. The finding of normocytic normochromic anaemia in bovine theileriosis agrees with the report of Ayadi et al. [34] for cattle reared in Algeria. The finding of a normal PCV value with a low red blood cell count and hemoglobin concentration in *A. platys* + *T. sinensis* is highly suggestive of the presence of large erythrocytes and a decrease in plasma volume due to dehydration, giving a false PCV value. Therefore, macrocytic normochromic anaemia reported in this cattle group is responsive because anisocytosis and macrocytic indices support the regenerative status in ruminants [75]. Degenerative left shift, hypernatremia, hyperkalemia, hypochloridemia, and hyperproteinemia with low albumin-to-globulin ratios were other hemato-biochemical features reported in *A. platys + T. sinensis* double species co-infection, and these were suggestive of an ongoing inflammatory condition and dehydration. The triple infection cattle group (*C. M. haemobos + T. sinensis + A. marginale*) had normal hematological values, but the presence of a few spherocytes on the blood smear may indicate possible recovery from immune-mediated hemolytic anaemia (IMHA) associated with blood pathogen infections. Most cases of IMHA in cattle have been associated with secondary infectious diseases such as theileriosis and anaplasmosis [76, 77], and a correlation between infection and autoimmunity was described based on the hypothesis of ‘molecular mimicry’. This mechanism suggests that there is an antigenic cross-reaction between pathogen-expressed epitopes, and self-molecules or autoantigens [78]. Animals infected with *Anaplasma* species produce antibodies against parasitized and non-parasitized erythrocytes [79]. Therefore, spherocytosis in IMHA usually occurs following partial phagocytosis of infected red blood cells in which altered cell membranes are coated with immunoglobulins (IgG and IgM) and complement (C3b) produced to combat the blood pathogens [24].

Increased globulin concentration in the *T. evansi-*, *C. M. haemobos-*, and *T. orientalis* + *A. marginale*-infected cattle groups signifies the presence of a current inflammatory condition caused by the bacteria and protozoa or by secondary bacterial infections. This is in agreement with the study by Ashuma et al. [80], who reported an increase in circulating immunoglobulins in dairy cattle naturally infected with *A. marginale*. Degenerative left shift was one of the major hematological abnormalities observed in all of the infected cattle groups, and it is a sign of severe acute inflammatory response to the blood pathogens. In this study, a degenerative left sign signifies that the demand for neutrophils from an inflammatory focus is overwhelming and that the granulopoietic capacity was exceeded [81, 82]. The functional storage compartment of mature neutrophils in the bone marrow of cattle is low when compared to other mammalian species [83], and in severe inflammatory conditions, increased demand for neutrophils rapidly exhausts the circulating and marrow storage pool. Subsequently, immature neutrophils such as the band, metamyelocytes, and myelocytes are released into peripheral circulation. Bacterial and protozoal infections tend to cause immunosuppression, which makes the host susceptible to secondary infections.

Natural infections of cattle with these blood pathogens increase the fragility of red blood cells. This suggests that, even when the blood pathogens are cleared or reduced from peripheral circulation, they ‘leave behind’ red blood cells that are susceptible to hypo-osmotic stress. It is physiologically expected that fewer RBCs should hemolyze at higher saline concentrations (0.9%), but in this case, more RBCs were still hemolyzed at this concentration, suggesting that the RBC membrane was compromised by the effect of the blood pathogens. Additionally, the EOF curves exhibited a rightward shift, and this further confirmed the increased instability of the red blood cell membrane induced by blood pathogens [84]. In general, blood pathogens induce structural, biochemical, and functional damage to the erythrocyte membrane (degradation of the membrane polyunsaturated fatty acids) and make them vulnerable to reactive oxygen species and susceptible to hemolysis [8, 37].

Our finding of hypernatremia in all cattle groups was not in agreement with the findings of Somu et al. [87], who reported hyponatremia in *Theileria*-infected cattle. Therefore, we suggest that a decrease in serum sodium level may have been present but was probably masked by dehydration. Prerenal dehydration could be responsible for the hyperkalemia and hyperchloridemia observed in all cattle groups [88]. The *Theileria orientalis, C. Mycoplasma haemobos,* and *Anaplasma marginale* triple infections confirmed by PCR tests in the sampled cattle test were considered subclinical based on the absence of anaemia, on the absence of blood pathogens on thin blood smears, on the presence of blood pathogen DNA, and on the presence of nonspecific clinical symptoms such as anorexia and ocular discharge. Therefore, not determining the different stages of blood pathogen infection (low, moderate, or high) is a limitation of the current study. Another limitation of this study is the failure to document the body conditions of the sampled cattle.

## Conclusions

Trypanosomosis, theileriosis due to *T. sinensis* and *A. platys + T. sinensis* mixed infections were observed to be the main causes of anaemia in 15 out of 61 (24.59%) sampled cattle. Therefore, we present the first evidence of *Theileria sinensis* associated bovine anaemia (TSABA) in Malaysian beef cattle. Anisocytosis, poikilocytosis, mild spherocytosis, degenerative left shift, increased plasma protein, hyperkalaemia, hypernatraemia, hyperchloridaemia were significant findings in most of the blood pathogen infected cattle groups. All infected cattle had circulating erythrocytes that are very susceptible to hypo-osmotic stress. Discovery of *Anaplasma platys* based on 16S rRNA gene detection in cattle raises a considerable concern about possible zoonotic transmission of this pathogen in Malaysia. Future research endeavours may incorporate species-specific primers targeting *A. platys* to confirm its current status in the ruminant industry, and design natural control and prevention measures to mitigate this issue facing Malaysian livestock sector. Future studies that aim to better understand the vector linked to *A. platys* transmission in Malaysian cattle and risk of exposure of *A. platys* to local farmers, veterinary and para-veterinary personnel was also recommended. Additional investigations are needed to determine which tick species serve as the main vector of *T. sinensis* in Malaysia. Quantification of the pathogen gene copy numbers to determine the stage of infection is also recommend for future research involving natural blood pathogen infections in cattle.

## Data Availability

DNA sequences obtained in this study have been submitted to GenBank database (accession number: MT184275-MT184279; MT271898 - MT271911; MT173810 - MT173814; MT259465 & MT259466; MT514513-MT514514; MT521752 - MT521753). All data generated and analysed during the study are included in this published work.

## Abbreviations

PCR: Polymerase chain reaction
TSABA: *Theileria sinensis* associated Bovine Anaemia
EDTA: Ethylene Diamine Tetraacetic Acid
MPSP: Major Piroplasm Surface Protein
MSP4: Major Surface Protein 4
rRNA: Ribosomal RNA
BLAST: Basic Local Alignment Search Tool
RoTat1.2 VSG: Rode Trypanozoon antigen type 1.2 variable surface glycoprotein
NCBI: National Center for Biotechnology Information
bp: Base pair
